# Characterization and expression analysis of transcription factors in *Spartina alterniflora* unveil their critical roles in salt stress resistance

**DOI:** 10.3389/fpls.2025.1592211

**Published:** 2025-08-21

**Authors:** Yuanyuan Jiang, Shoukun Chen, Shuqiang Gao, Jiahui Geng, Qin Shu, Shang Gao, Huihui Li

**Affiliations:** ^1^ School of Tropical Agriculture and Forestry, Hainan University, Haikou, China; ^2^ Nanfan Research Institute, Chinese Academy of Agricultural Sciences (CAAS), Sanya, Hainan, China; ^3^ State Key Laboratory of Crop Gene Resources and Breeding, Institute of Crop Sciences, Chinese Academy of Agricultural Sciences (CAAS), Beijing, China; ^4^ Guangxi Key Laboratory of Rice Genetics and Breeding, Rice Research Institute, Guangxi Academy of Agricultural Sciences, Nanning, Guangxi, China

**Keywords:** expression patterns, genome-wide, salt stress, *Spartina alterniflora*, transcription factor

## Abstract

**Introduction:**

Transcription factors (TFs) are essential regulators of gene expression, orchestrating plant growth, development, and responses to environmental stress. *Spartina alterniflora*, a halophytic species renowned for its exceptional salt resistance, provides an ideal model for investigating the regulatory mechanisms underlying salt tolerance.

**Methods:**

Here, we present a comprehensive genome-wide identification and characterization of TFs in *S. alterniflora*. A total of 5,004 TFs were identified and classified into 56 families, with bHLH, MYB, NAC, and ERF being the most abundant. Gene structure analysis revealed an average of 5.05 exons per TF, with significant variation in exon number, coding sequence length, and GC content across families, reflecting their structural and functional diversity. Evolutionary analysis indicated that *S. alterniflora* TFs have undergone gene duplication events, with purifying selection (*Ka*/*Ks* < 1) shaping their evolution. Tissue-specific expression analysis revealed distinct TF expression patterns across roots, stems, leaves, inflorescences, and seeds, underscoring their roles in organogenesis. Under salt stress, 800 TFs exhibited differential expression, with MYB, bHLH, bZIP, ERF, and NAC families being the most responsive, suggesting their involvement in ion homeostasis, osmoregulation, and antioxidant defense.

**Results and Discussion:**

These findings provide key insights into the transcriptional regulation of salt resistance in *S. alterniflora*, offering valuable genetic targets for enhancing crop resilience to salinity.

## 1 Introduction

Transcription factors (TFs) play a central role in regulating gene expression, governing diverse biological processes, including growth, development, and stress adaptation. Among abiotic stresses, salinity poses a major challenge to plant survival and productivity, particularly in the context of climate change and soil salinization. The proportion of TFs in plant genomes varies, with *Arabidopsis thaliana* has approximately 2,300 TFs (~8.3% of its total genes) (Jin et al., 2017), while crop species exhibit similar proportions, including wheat (*Triticum aestivum*, 5.7%), rice (*Oryza sativa*, 6.5%), foxtail millet (*Setaria italica*, 7.4%), and rapeseed (*Brassica napus*, 6.1%) (Priya and Jain, 2013; Zheng et al., 2016; Jin et al., 2017; Evans et al., 2022). Notably, plant genomes generally contain a higher proportion of TFs than comparably sized animal genomes, such as *Drosophila melanogaster* (5.5%) (Pfreundt et al., 2010; Shokri et al., 2019), mouse (*Mus musculus*, 6.8%), and maize (*Zea mays*, 8.3%) (Jin et al., 2017; Zhou et al., 2017), suggesting that TFs play a particularly critical role in plant-specific regulatory networks (Shiu et al., 2005; Mitsuda and Ohme-Takagi, 2009).

As primary regulators of gene expression, TFs influence the proteome, metabolome, and phenome (Casamassimi and Ciccodicola, 2019), which enables the functional specialization of cells with identical genetic material (Weidemüller et al., 2021). They govern key developmental processes, including floral trait formation (Sasaki, 2018), fruit morphology (Seymour et al., 2013), thermomorphogenesis (Delker et al., 2022), organogenesis, and symbiotic nodulation in legumes (Motte et al., 2019; Gong et al., 2020; Verma et al., 2022; Su et al., 2023). Moreover, TFs regulate signal transduction, stress responses, and metabolic pathways, underscoring their broad functional significance (Ruan, 2014; Jiang et al., 2017; Gong et al., 2020; Javed and Gao, 2023).

*Spartina alterniflora*, commonly known as smooth cordgrass, is a halophyte adapted to high-salinity environments such as coastal marshes and estuaries (Chelaifa et al., 2010). Its extreme salt resistance makes it an excellent model for investigating the molecular mechanisms underlying plant resistance to saline conditions (Yang et al., 2023; Chen et al., 2024; Huang et al., 2024). TFs play a pivotal role in regulating downstream genes involved in immediate physiological response to salt stress, mediating physiological and biochemical responses such as ion homeostasis, osmotic adjustment, and antioxidant defense (Weidemüller et al., 2021). However, despite its prominence as a halophyte model, the TF repertoire of *S. alterniflora* remains entirely unexplored. Crucially, this knowledge gap impedes our understanding of the transcriptional regulatory basis of its extreme halophytism. Most research focuses on model plants like *A. thaliana* or staple crops like rice, which exhibit comparatively lower salt resistance. Thus, a genome-wide characterization of *S. alterniflora* TFs and their expression dynamics under salt stress is essential to uncover the unique regulatory networks underpinning its exceptional resilience.

Given the increasing global threat of soil salinization, the identification of salt-responsive TFs in halophytes like *S. alterniflora* represents a significant step toward improving agricultural sustainability in salt-affected regions. This study aims to address this gap by conducting a genome-wide analysis of *S. alterniflora* TFs, integrating transcriptomic and phylogenetic analyses to elucidate their roles in salt resistance. Our findings will not only enhance the understanding of transcriptional regulation in halophytes but also provide a foundation for genetic engineering and breeding strategies to improve crop resilience to salinity. Ultimately, this research contributes to leveraging halophyte genetics to mitigate the impacts of climate change and soil degradation, supporting global food security and sustainable agriculture.

## 2 Materials and methods

### 2.1 Genome-wide identification of TFs in *S. alterniflora*

To identify TFs in *S. alterniflora*, we first retrieved TF protein sequences from rice, maize, and *Arabidopsis* in the PlantTFDB database (Jin et al., 2017). Using these sequences, we constructed hidden Markov models (HMMs) for each TF family and screened the *S. alterniflora* proteome with HMMSearch, applying an *E*-value threshold of < e^-5^. In parallel, we performed a BLASTP search against the *S. alterniflora* reference genome using TF protein sequences from rice, wheat, maize, and *Arabidopsis* as queries, with thresholds set at E*-*value < e^-5^ and sequence identity > 80%. To ensure the accuracy of candidate TFs, we verified the presence of conserved domains using NCBI Conserved Domains Database (NCBI-CDD) (Marchler-Bauer et al., 2015), retaining proteins with domain coverage > 50%. Redundant sequences were manually curated to finalize the TF dataset. The genomic and annotation data for *Spartina alterniflora* are available in our previously published study (Chen et al., 2024) and the GenBank under the accession number JARYIK000000000.

### 2.2 Gene ontology enrichment analyses

TFs were functionally annotated using NetGO 3.0 (Wang et al., 2023), following default program parameters. Enrichment analysis was conducted to identify significantly overrepresented gene sets, using Fisher’s exact test (*P* < 0.05) and an enrichment threshold of ≥ 1.5-fold relative to the genomic background.

### 2.3 Phylogenetic analysis

Full-length protein sequences of each TF family were aligned using MUSCLE v3.8.31 (Edgar, 2004) implemented in MEGA7 (Kumar et al., 2016) with the following optimized parameters: Gap Open = -2.9, Gap Exten = 0, Hydrophobicity Multiplier = 1.2. For phylogenetic reconstruction, the Neighbor-Joining (NJ) method was employed in MEGA7 using the Bootstrap method. Model selection was validated using the Poisson model implemented in MEGA’s model selection tool. The tree topology was evaluated with 1,000 non-parametric bootstrap replicates to assess node reliability. The final tree was visualized using Evolview (Subramanian et al., 2019).

### 2.4 Detection of gene duplication events

Gene duplication events were identified using MCScanX (Wang et al., 2012). Full-length transcripts from *S. alterniflora* were subjected to collinearity analysis in MCScanX, using BLASTP (v2.8.1) with the following parameters: -evalue e^-10^ -num_alignments 5 -outfmt 6. Non-synonymous (Ka) and synonymous (Ks) substitution rates, along with Ka/Ks ratios, were estimated for each TF family using TBtools (v2.154) (Chen et al., 2020).

### 2.5 GC content estimation

GC content was calculated as the percentage of guanine and cytosine bases relative to the total sequence length for each TF gene family. Analyses were performed using SeqKit (Shen et al., 2016).

### 2.6 Gene expression trend analysis

For RNA-seq data analysis, raw reads were processed by adapter trimming and quality filtering to generate clean reads, which were then aligned to the *S. alterniflora* reference genome using HISAT2 (Kim et al., 2019). Gene expression levels were quantified as FPKM (fragments per kilobase of exon per million mapped fragments) using uniquely mapped reads via featureCounts (Liao et al., 2014). Differential expression analysis was performed with DESeq2 (Love et al., 2014), identifying DEGs using thresholds of |log_2_(fold change)| ≥ 2 and an FDR-adjusted *p*-value < 0.01. Subsequently, trend analysis categorizing genes with similar expression patterns was conducted using the Short Time-series Expression Miner (STEM) tool on the OmicShare platform (www.omicshare.com/tools). This analysis focused on significantly enriched expression profiles (*p* < 0.05), with nine distinct trends selected for investigation. The raw data pertaining to different tissues and various concentrations of NaCl treatment are available under the SRA accession numbers PRJNA949976 and PRJNA1105679, respectively.

### 2.7 RNA extraction and quantitative real-time PCR analysis

To investigate the expression dynamics of TF genes under salt stress, we subjected *S. alterniflora* plants to six NaCl concentration gradients (0, 100, 200, 300, 500, and 700 mM) for a duration of two months. Total RNA was extracted from the leaves of these salt-stressed plants using the Plant RNA Purification Reagent (TIANGEN, Beijing, China), strictly adhering to the manufacturer’s protocol. First-strand cDNA was synthesized via reverse transcription using the Evo M-MLV Reverse Transcriptase Kit (Accurate Biology, Changsha, China). The synthesized cDNA was diluted 10-fold and served as the template for qRT-PCR analysis. Specific primers were designed using Oligo 7.0 software, and qRT-PCR was performed using the SYBR Green kit (Accurate Biology, Changsha, China), strictly adhering to the manufacturer’s protocol. Each treatment included three biological replicates, and relative gene expression levels were calculated using the 2^-ΔΔCt^ method. Data were analyzed and visualized using Excel 2010, and statistical significance was assessed using the least significant difference (LSD) test (*p* ≤ 0.05).

## 3 Results

### 3.1 Genome-wide identification of TFs in *S. alterniflora*

Using Hidden Markov Models (HMMs) tailored for various transcription factor (TF) families, we identified 5,004 TFs in the *S. alterniflora* genome. These TFs are distributed across all 31 chromosomes, with the highest numbers found on chromosomes 1, 2, and 9, which harbor 261, 284, and 273 TFs, respectively (**Supplementary Table S1**). In contrast, chromosome 31 has the lowest count, with only 52 TFs. Based on their structural characteristics, these TFs were classified into 56 distinct families. Among them, the bHLH (basic helix-loop-helix), MYB (Myeloblastosis), NAC (NAM, ATAF1/2, and CUC2), and ERF (ethylene-responsive factor) families are the most abundant, comprising 469, 399, 333, and 318 members, respectively. Conversely, families with fewer than five members include NF-X1 (Nuclear Factor-X1, 4), S1Fa-like (3), STAT (Signal Transducer and Activator of Transcription, 3), and HRT-like (High Responsiveness to Temperature-like, 2).

**Figure 1:**
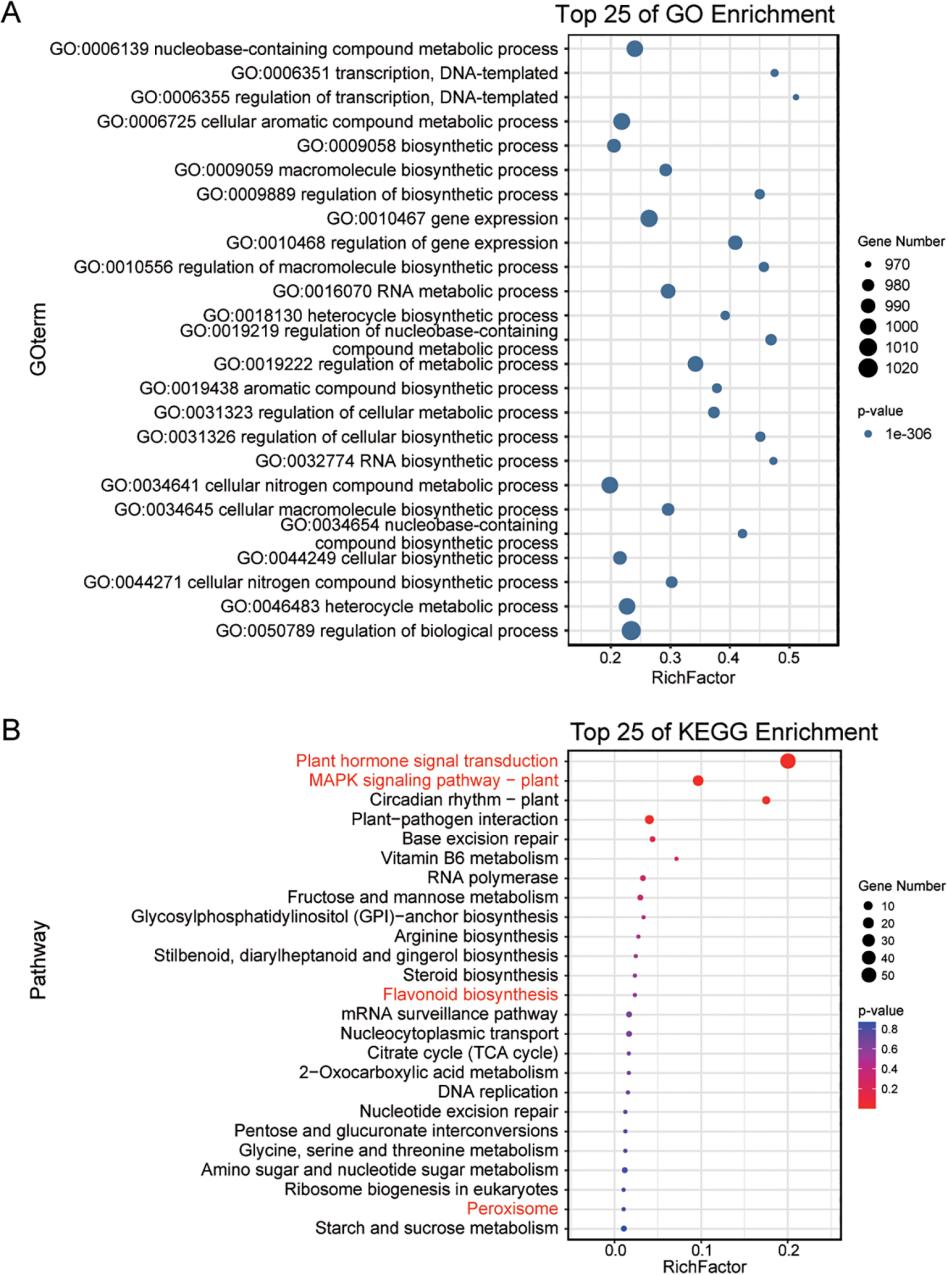
Functional enrichment analysis of TFs in *S. alterniflora*. **(A, B)** display the Gene Ontology (GO) and Kyoto Encyclopedia of Genes and Genomes (KEGG) enrichment analyses of TFs in *S. alterniflora*, respectively. These analyses highlight the top 25 most enriched biochemical pathways associated with these TFs.

Gene Ontology (GO) enrichment analysis of TFs revealed significant enrichment in transcriptional regulation processes (**Figure 1A**), including transcription (GO: 0006351), regulation of transcription (GO: 0006725), gene expression (GO: 0010467), and regulation of gene expression (GO: 0010468). Additionally, these TFs are associated with key metabolic and biosynthetic pathways, such as biosynthetic process (GO: 0009058), regulation of biosynthetic process (GO: 0009889), and regulation of biological process (GO: 0050789). Kyoto Encyclopedia of Genes and Genomes (KEGG) pathway analysis further indicated enrichment in pathways relevant to salt stress resistance (**Figure 1B**), including plant hormone signal transduction, MAPK (Mitogen-Activated Protein Kinase) signaling, flavonoid biosynthesis, and peroxisome function.

### 3.2 Gene structure and GC content analysis

Analysis of TF gene structures provides insights into their evolutionary trajectories. The average exon count across all TF genes is 5.05, with the ZF-HD (Zinc Finger-Homeodomain) family exhibiting the fewest exons (1.73 per gene) and the CAMTA (Calmodulin-Binding Transcription Activator) family the most (13.24 per gene). Among the 5,004 identified TFs, 2,322 genes contain both 5’ and 3’ untranslated regions (UTRs), while 281 genes possess only a 5’UTR, 445 have only a 3’UTR, and 1,956 lack UTRs altogether (**Supplementary Table S1**), reflecting considerable structural diversity.

Further investigation into coding sequence (CDS) lengths revealed that 34 TF families have average CDS lengths between 1,000 and 2,000 bp, while 16 families have CDS lengths below 1,000 bp, and five exceed 2,000 bp. Notably, the STAT family has the longest average CDS length (3,653 bp), whereas the S1Fa-like family has the shortest (654 bp) (**Supplementary Table S1**). These differences likely reflect functional specializations and evolutionary adaptations.

GC content analysis of TF coding sequences uncovered substantial variation among TF families. The SRS family exhibits the highest GC content (69.59%), and the SRS family members are distributed across multiple chromosomes, including 01G, 04G, 06G, 03G, 05G (with two members), 13G (with two members), 14G (with two members), 15G, 16G, 18G, 19G, 23G, 24G (with two members), 17G, and 20G. Meanwhile, the HB-PHD (Homeobox-PHD finger) family has the lowest (44.16%) (**Supplementary Table S1**). These differences may influence genomic stability, transcriptional regulation, and interactions with other biomolecules, ultimately shaping the function and evolution of TFs in *S. alterniflora*.

### 3.3 Evolutionary expansion and duplication of TFs in *S. alterniflora*

**Figure 2:**
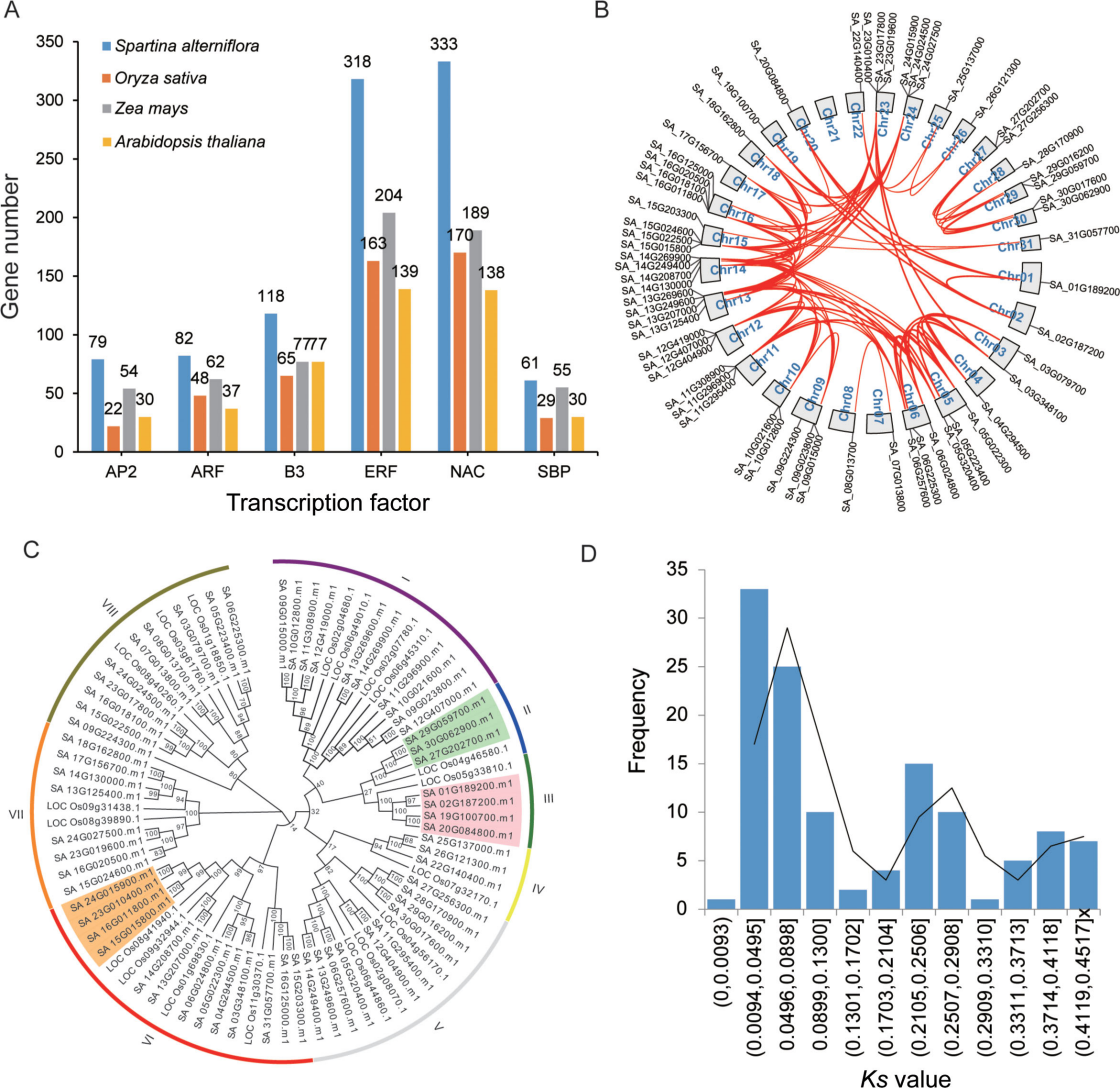
Evolutionary duplication of SBP TFs in *S. alterniflora*. **(A)** Proportion of plant-specific TFs in *S. alterniflora*, rice, maize, and *Arabidopsis*. **(B)** Distribution of SBP TFs across the *S. alterniflora* genome, with red lines connecting duplicated genes. **(C)** Phylogenetic comparison of SBP TFs between *S. alterniflora* and rice using MEGA7. **(D)** Distribution of synonymous substitution rates (Ks) among collinear paralogs in *S. alterniflora*.

To further investigate the evolutionary expansion and duplication events of TFs, we conducted a genome-wide identification of TF numbers in various species including *S. alterniflora* (**Supplementary Table S2**). The results revealed that *S. alterniflora* possesses 5,004 TFs, which is fewer than the 6,148 identified in Glycine max but exceeds the counts in *Oryza sativa* (2,408), *Zea mays* (3,308), *Setaria italica* (2,410), and *Arabidopsis thaliana* (2,294). For a deeper evolutionary analysis of TF dynamics in *S. alterniflora*, we examined plant-specific TF families including AP2 (APETALA2), ERF, NAC, WRKY, B3, ARF (AUXIN RESPONSE FACTOR), and SBP (SQUAMOSA PROMOTER BINDING PROTEIN). Our findings indicate that these plant-specific TFs have undergone genomic-level expansion in *S. alterniflora* compared to the other analyzed species (**Figure 2A**; **Supplementary Table S2**). For instance, the SBP TF family consists of 61 members, representing expansions of 2.10-, 1.11-, and 2.03-fold relative to rice, maize, and *Arabidopsis*, respectively. This suggests that SBP TFs have undergone significant duplication events during *S. alterniflora* evolution. Intragenomic collinearity analysis revealed strong synteny among SBP TF genes, with 60 SBP genes forming 121 collinear gene pairs (**Figure 2B**). Synonymous substitution rate (Ks) analysis identified a bimodal distribution of paralogs, with peaks at Ks ~0.050 and ~0.251, indicative of at least two whole-genome duplication (WGD) events. These findings support the hypothesis that gene duplication contributed to SBP TF expansion in *S. alterniflora*. Phylogenetic comparisons with rice further confirm expansion within specific SBP subgroups. For example, Group II contains three *S. alterniflora SBP* genes and only one rice homolog. In comparison, Group III consists of four *S. alterniflora SBP* genes and a single rice *SBP* gene (**Figure 2C**), providing further evidence of lineage-specific expansion.

In addition, we constructed phylogenetic trees for ARF, NAC, WRKY, AP2, and RAV families (with RAV representing a distinct subclade within the B3 superfamily). Our analysis revealed that, similar to the SBP TF family, these TF families have undergone continuous expansion during evolutionary history (**Supplementary Figure S1**). Taking the AP2 TF family as an example, we identified a clade in Group I containing ten *S. alterniflora* AP2 TFs and a single rice AP2 TF. Within Group I of the RAV TF family, we observed eight *S. alterniflora* RAV homologs compared to only two rice counterparts. These findings provide further evidence of lineage-specific expansion events in these TF families, particularly in the halophytic species *S. alterniflora*.

To assess selective pressures acting on these TFs, we calculated the Ka/Ks ratio for duplicated gene pairs. Using MCscanX, we identified 3,429 TFs forming 8,603 duplicated pairs. Notably, all duplicated gene pairs exhibited Ka/Ks ratios below 1 (**Figure 2D**; **Supplementary Table S3**), indicating that *S. alterniflora* TFs have predominantly evolved under purifying selection, preserving their functional integrity while undergoing expansion.

### 3.4 Analysis of TF gene salt stress responses in *S. alterniflora*

**Figure 3:**
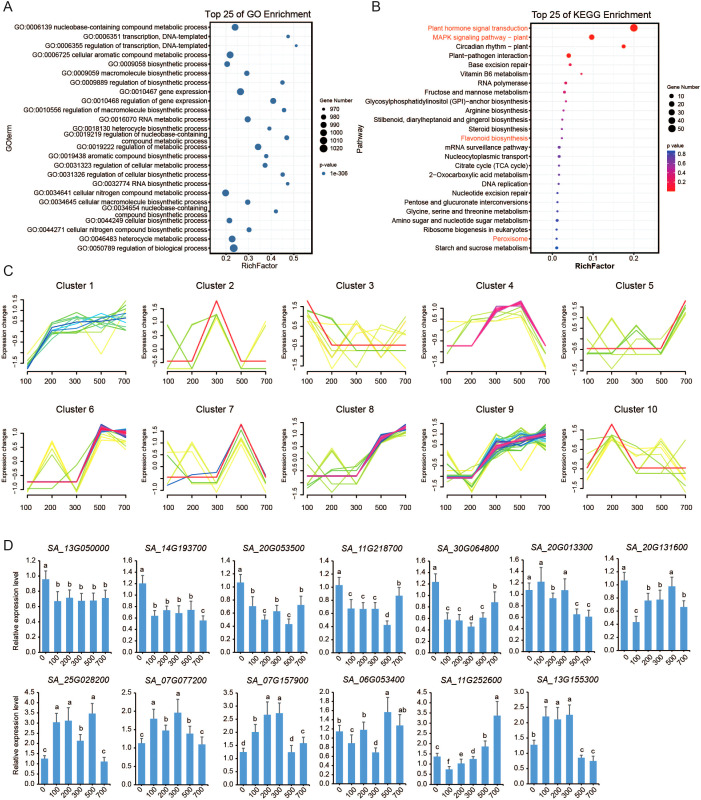
Differential expression of TF genes induced by salt stress. **(A)** GO enrichment analysis of differentially expressed TF genes under salt stress. **(B)** KEGG analysis of differentially expressed TF genes, showcasing the top 25 most enriched biochemical pathways. **(C)** Trend expression analysis categorizes the differentially expressed TFs into 10 distinct expression profiles, revealing salt stress-specific regulatory patterns. The x-axis represents various NaCl concentrations, and the y-axis indicates relative changes in gene expression levels (log_2_ fold change value) for each TF profile. **(D)** Relative expression levels of the 13 TF genes following treatment with various concentrations of NaCl are shown. Error bars represent the standard error of the mean (SEM; n = 3). Columns labeled with different letters indicate statistically significant differences (*p* ≤ 0.05).

To assess the function of TF genes under salt stress, we prioritized the identification of key regulators by first screening for TF genes exhibiting significant different expression (|log_2_FC| ≥ 1) across five NaCl concentration. A total of 800 TF genes were differentially expressed. With the exception of LFY (LEAFY), NF-X1, S1Fa-like, STAT, and Whirly family members, at least one gene from every other TF family was responsive to salt stress. The MYB, bHLH, bZIP, ERF, and NAC families exhibited the highest numbers of differentially expressed genes. Gene Ontology (GO) enrichment analysis revealed that these genes were functionally categorized similarly to the overall TF gene set (**Figures 3A, B**), emphasizing their essential regulatory roles in stress response process, growth, and metabolic pathways (**Figure 1**). Specifically, these differentially expressed TFs were significantly enriched in transcriptional regulation, gene expression control, and metabolic processes, further supporting their central role in environmental stress response.

To refine our understanding of these 800 TF genes, we classified their expression profiles into three main trends (**Figure 3C**): continuously upregulated (clusters 1, 8, and 9), intermediately upregulated (clusters 2, 4, 7, and 10), and irregularly changed (clusters 3, 5, and 6). Notably, 13 TF genes were differentially expressed under all salt treatment conditions. Among them, seven genes were continuously downregulated, including *SA_13G050000.m1* and *SA_14G193700.m1* from the AP2 family, *SA_20G053500.m1* from the B3 family, *SA_11G218700.m1* and *SA_30G064800.m1* from the ERF family, *SA_20G013300.m1* from the MYB-related family, and *SA_20G131600.m1* from the NAC family. Conversely, three genes were continuously upregulated, including *SA_25G028200.m1* from the G2-like (GATA-2 like) family, *SA_07G077200.m1* from the LBD family, and *SA_07G157900.m1* from the MYB family. The remaining three genes exhibited variable expression patterns: *SA_06G053400.m1* from the ERF family was upregulated at 200 mM NaCl but downregulated under other conditions; *SA_11G252600.m1* from the G2-like family was downregulated at 100 mM NaCl but upregulated otherwise, and *SA_13G155300.m1* from the WRKY family was downregulated at 700 mM NaCl while being upregulated under other conditions.

We further analyzed the expression patterns of these 13 TF genes using qRT-PCR (**Figure 3D**). The results revealed that all 13 genes exhibited altered expression in response to varying NaCl concentrations. Among them, five genes (*SA_13G050000.m1*, *SA_14G193700.m1*, *SA_20G053500.m1*, *SA_11G218700.m1*, and *SA_30G064800.m1*) showed significant downregulation under NaCl treatment. The remaining eight genes displayed irregular expression patterns under NaCl treatment. Comparison with transcriptome data demonstrated that ten genes exhibited consistent expression trends between qRT-PCR and RNA-seq, while *SA*_*20G013300.m1*, *SA*_*06G053400.m1*, and *SA*_*13G155300.m1* showed discordant results.

### 3.5 Analysis of tissue expression patterns of TF genes in *S. alterniflora*

Building on the salt-responsive TF cohort identified in salt-responsive, we analyzed their tissue-specific expression profiles across roots, stems, young and mature leaves, developing inflorescences, mature seeds, and germinating seeds to understand how these regulators are deployed in salt-affected tissues. A total of 392 TF genes showed no detectable expression, while the remaining genes were expressed in at least one tissue. Notably, all members of 18 TF families (BBR-BPC (Barley B Recombinant/Basic Pentacysteine), CAMTA, CO-like (CONSTANS-like), CPP (Cysteine-Rich Polycomb-Like Protein), DBB (Double B-box zinc finger), GRF (Growth-Regulating Factor), HB-PHD, HRT-like, HSF (Heat Shock Transcription Factor), LSD (Lateral Organ Boundaries-Domain), NF-YA (Nuclear Factor-Y subunit A), NF-YC (Nuclear Factor-Y subunit C), RAV (Related to ABI3/VP1), S1Fa-like (S1Fa-like transcription factor), SRS (SHI-Related Sequence), STAT (Signal Transducer and Activator of Transcription), VOZ (Vascular plant One-Zinc finger), and Whirly) were detected in at least one tissue, indicating their broad transcriptional activity.

**Figure 4:**
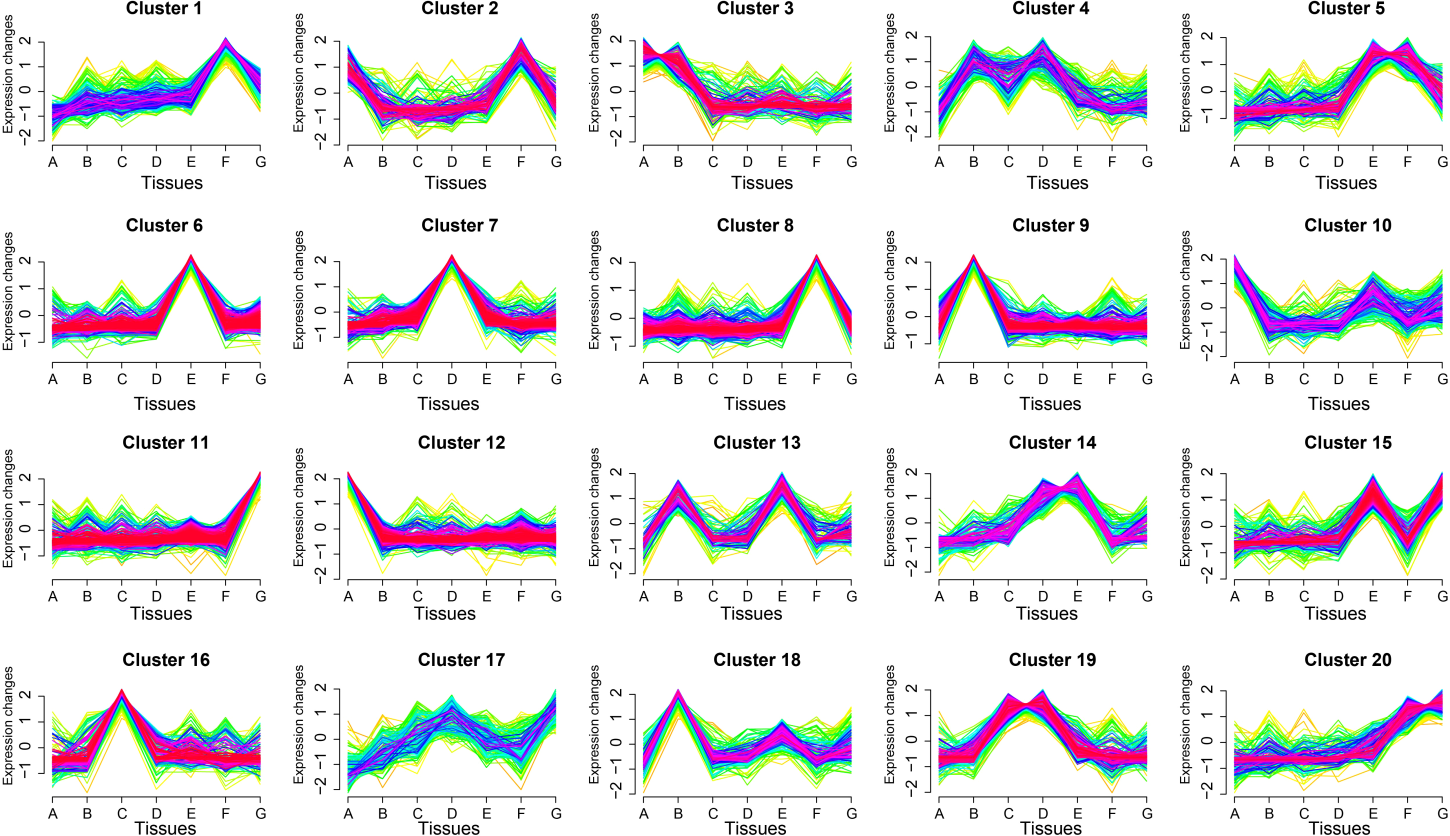
Expression trend analysis of TF genes across different tissues of *S. alterniflora*. The x-axis represents various tissues: roots **(A)**, stems **(B)**, young leaves **(C)**, mature leaves **(D)**, developing inflorescences **(E)**, mature seeds **(F)**, and germinating seeds **(G)**. The y-axis shows the changes in gene expression trends, with 20 distinct expression patterns depicted. Each profile highlights tissue-specific or developmentally regulated expression trends, providing insights into the functional roles of TF genes in *S. alterniflora* growth, development, and adaptation. The analysis depicted in the figure was performed using the FPKM (Fragments Per Kilobase of transcript per Million mapped reads) values of each gene across different tissues.

To identify tissue-specific expression patterns, we employed the Short Time-series Expression Miner (STEM) program to cluster genes with similar expression profiles (**Figure 4**). A total of 20 distinct clusters were identified. Cluster 11 contained the most genes (498), followed by cluster 6 (485) and cluster 8 (387). Several clusters exhibited clear tissue specificity: cluster 1 and cluster 8 genes were predominantly expressed in mature seeds; cluster 6 genes were highly expressed in developing inflorescences; cluster 7 genes showed peak expression in mature leaves; cluster 9 genes were predominantly expressed in stems; cluster 11 genes were primarily active in germinating seeds; cluster 12 genes were highly expressed in roots; and cluster 16 genes were most abundant in young leaves. These findings provide critical insights into the functional roles of TFs in *S. alterniflora* development.

Further analysis revealed that multiple gene expression modules displayed tissue-specific expression patterns. For example, cluster 2 genes were enriched in roots and mature seeds, cluster 3 genes were prominent in roots and stems, and cluster 4 genes showed high expression in stems and mature leaves. Cluster 5 genes were active in developing inflorescences and mature seeds, while cluster 10 genes were found in roots and developing inflorescences. Other notable clusters included cluster 13 (stems and developing inflorescences), cluster 14 (mature leaves and developing inflorescences), cluster 15 (developing inflorescences and germinating seeds), cluster 17 (mature leaves and germinating seeds), cluster 19 (young and mature leaves), and cluster 20 (mature seeds).

We further investigated the enrichment of major TF families within each expression module. In addition to the widespread presence of bHLH, bZIP (Basic Leucine Zipper), C2H2 (Cys_2_-His_2_ zinc finger), ERF, MYB, NAC, and WRKY families, other TF families were also detected in at least one cluster. To identify key regulators, we examined the dominant gene families in each cluster. MYBs accounted for the highest proportion in clusters 3 (14%), 9 (21%), and 18 (11%), suggesting a primary role in root and stem development. WRKYs were most abundant in clusters 7 (18%) and 14 (12%), indicating their involvement in mature leaves and inflorescences. The bHLH family was highly represented in clusters 4, 5, 6, 12, and 16, suggesting a broad role in *S. alterniflora* growth and development.

Given that *Spartina alterniflora* roots are directly exposed to saline environments under natural conditions, and numerous studies have established the root system as the primary sensor of salt stress orchestrating early stress responses through mechanisms such as ion compartmentalization, osmotic adjustment, and hormone signaling transduction (Ibeas et al., 2024; Lu et al., 2024), we conducted an in-depth analysis of root-specific cluster 12. This cluster comprises 330 TF genes, with 39 (11.8%) exhibiting significant differential expression under salt treatment (|log_2_FC| ≥ 1, FDR < 0.05). Notably, a greater number of DEGs were observed under extreme saline conditions (500 and 700 mM NaCl; **Supplementary Figure S2**), suggesting potential involvement in terminal mechanisms for high-salinity resistance.

Functional enrichment analysis revealed significant overrepresentation of this gene subset in key salt-tolerance pathways, including “Plant hormone signal transduction” and “MAPK signaling pathway”. Further TF family classification showed that bHLH, HD-ZIP, MYB_related, and WRKY families constituted the largest proportions of DEGs. Notably, the MYB_related TF SA_16G171600 exhibits high homology with rice *CIRCADIAN CLOCK-ASSOCIATED1* (*OsCCA1*) and soybean *GmMYB133*. Previous studies demonstrate that *OsCCA1* enhances salt tolerance by regulating Na^+^/K^+^ homeostasis and ABA signaling (Li et al., 2024), while *Arabidopsis* overexpressing *GmMYB133* exhibits improved seed germination and plant growth under salt stress, accompanied by increased chlorophyll content and reduced malondialdehyde (MDA) accumulation (Shan et al., 2021).

## 4 Discussion

The expansion of TF families is a crucial evolutionary strategy that enables plants to withstand environmental stresses (Lehti-Shiu et al., 2017). In *S. alterniflora*, TF families such as bHLH (basic Helix-Loop-Helix, regulating diverse processes including stress response and development through dimerization) (Gao and Dubos, 2024), MYB (Myeloblastosis, characterized by DNA-binding domains involved in development, metabolism, and abiotic stress responses) (Bhatt et al., 2025), NAC (NAM, ATAF1/2, CUC2, crucial for development and stress signaling) (Xiong et al., 2025), and ERF (Ethylene Response Factor, key in hormone and stress responses) (Su et al., 2025) contain significantly more members than those in rice, maize, or *Arabidopsis*. This expansion provides additional regulatory mechanisms for coping with high-salinity environments. Critically, salt resistance in *Spartina* involves overlapping responses to osmotic imbalance, ion toxicity, and oxidative stress (Hessini et al., 2008; Hessini, 2022), necessitating coordinated TF regulation. Through whole-genome duplication (WGD) and gene duplication (Chen et al., 2024), *S. alterniflora* has acquired a larger repertoire of TF genes, which have been subjected to purifying selection (Ka/Ks < 1), likely preserving their functional integrity. However, while gene duplication is a recognized evolutionary driver, its specific contribution to halophyte evolutionary adaptation—compared to glycophytes—requires deeper mechanistic validation beyond copy number comparisons. Notably, the expansion of the *S. alterniflora* TFs family, such SBP, NAC, WRKY, AP2, RAV, and ARF, in *S. alterniflora* is hypothesized to enhance its salt resistance. These genomic features collectively enable *S. alterniflora* to thrive in saline niches, yet the functional redundancy versus specialization of duplicated TFs remains a critical unresolved question in the field (Mansour and Hassan, 2022; Lee, 2023; Chen et al., 2024).

Gene evolution in *S. alterniflora* involves functional diversification, as seen in expanded families like SBP, NAC, WRKY, AP2, RAV, and ARF, have undergone significant expansion through gene duplication. Gene duplication not only increases gene copy numbers but also promotes functional specialization through subfunctionalization or neofunctionalization, allowing duplicated genes to acquire novel roles (Rastogi and Liberles, 2005). Some genes regulate responses to environmental stresses, while others influence growth and development. This functional partitioning may be essential for managing the concurrent stress responses induced by salinity—including ion homeostasis disruption and oxidative damage—observed in expression datasets. In the B3 family, for example, the duplicated gene pair *SA_01G212000.m2* and *SA_20G053500.m1* exhibits distinct expression patterns: the former shows no differential expression under salt stress, while the latter is significantly downregulated under all tested NaCl concentrations. Similarly, in the bHLH family, *SA_03G020600.m1* remains unresponsive to salt stress, whereas its paralog *SA_06G318000.m1* is strongly upregulated at NaCl concentrations ranging from 200 to 700 mM. A contrasting pattern is also observed in the GATA family, where *SA_01G000700.m1* is barely detectable in any tissue, while *SA_02G001700.m1* exhibits high expression in roots, developing inflorescences, mature seeds, and germinating seeds. These cases suggest functional divergence fine-tunes stress-responsive networks. However, the direct physiological impact of these expression differences—beyond correlation—needs validation through mutagenesis or knockdown studies.

The expansion of TF genes in *S. alterniflora*, including key regulators like MYB (involved in abiotic stress signaling) and bHLH (often forming complexes with MYB TFs), has likely contributed to the establishment of complex regulatory networks essential for salt stress adaptation. For example, the orthologs of trichome-related AtR3-MYB genes, *SaCPC1* and *SaCPC2*, were identified in *S. alterniflora*, which may be involved in salinity responses (Gao et al., 2023). And SaMYB35 has been identified as a key regulator in the flavonoid biosynthesis pathway under salt stress conditions (Chen et al., 2025). When overexpressed in rice (cv. ZH11) grown under high salinity, SaMYB35 induces the upregulation of critical flavonoid biosynthetic genes, leading to increased flavonoid accumulation and enhanced salt tolerance compared to wild-type plants. In this study, TF families such as MYB, bHLH, and NAC exhibited co-expression patterns under salt stress, suggesting their coordinated roles in regulating downstream gene expression. Homologs of these TFs in crops have been implicated in enhancing salt tolerance. For instance, *OsMYB2* modulates the expression of the amino acid transporter *OsANT1*, thereby influencing rice growth and salt tolerance (Nie et al., 2025). The high-affinity potassium transporter OsHKT1;1, which plays a critical role in salt tolerance, is regulated by an MYB TF (Wang et al., 2015). Furthermore, the heterologous expression of *ZmbHLH36* in plants enhances salt tolerance by reducing malondialdehyde (MDA) accumulation and increasing peroxidase activity (Dai et al., 2024). Similarly, *OsNAC1* positively regulates drought and salt tolerance by directly participating in abscisic acid biosynthesis (Ao et al., 2024). These findings highlight the functional significance of TFs in regulating salt tolerance, offering theoretical insights and genetic resources for stress-resistant crop breeding. Yet, halophyte-specific innovations—such as neofunctionalized duplicates absent in crops—warrant further exploration to distinguish conserved versus novel mechanisms.

Tissue-specific TF expression in *S. alterniflora* (e.g., MYB in roots/stems, WRKY in leaves/inflorescences) tailors stress responses across organs. This mirrors findings in other halophytes: *Limonium bicolor* LbTRY alters root development (Leng et al., 2021), *Populus* PagMYB205 impairs ROS scavenging (Zhou et al., 2023), and birch BpWRKY32 enhances osmotic adjustment (Liu et al., 2024). However, *S. alterniflora*’s 800 differentially expressed TFs under salt stress likely regulate ion homeostasis, osmotic balance, and antioxidant defense. The overlap between salt-responsive and oxidative stress genes underscores interconnected stress-response pathways. While TF expansion is adaptive, the energy cost of maintaining large TF families in resource-limited environments presents an evolutionary trade-off requiring investigation (Wu et al., 2021).

**Figure 5:**
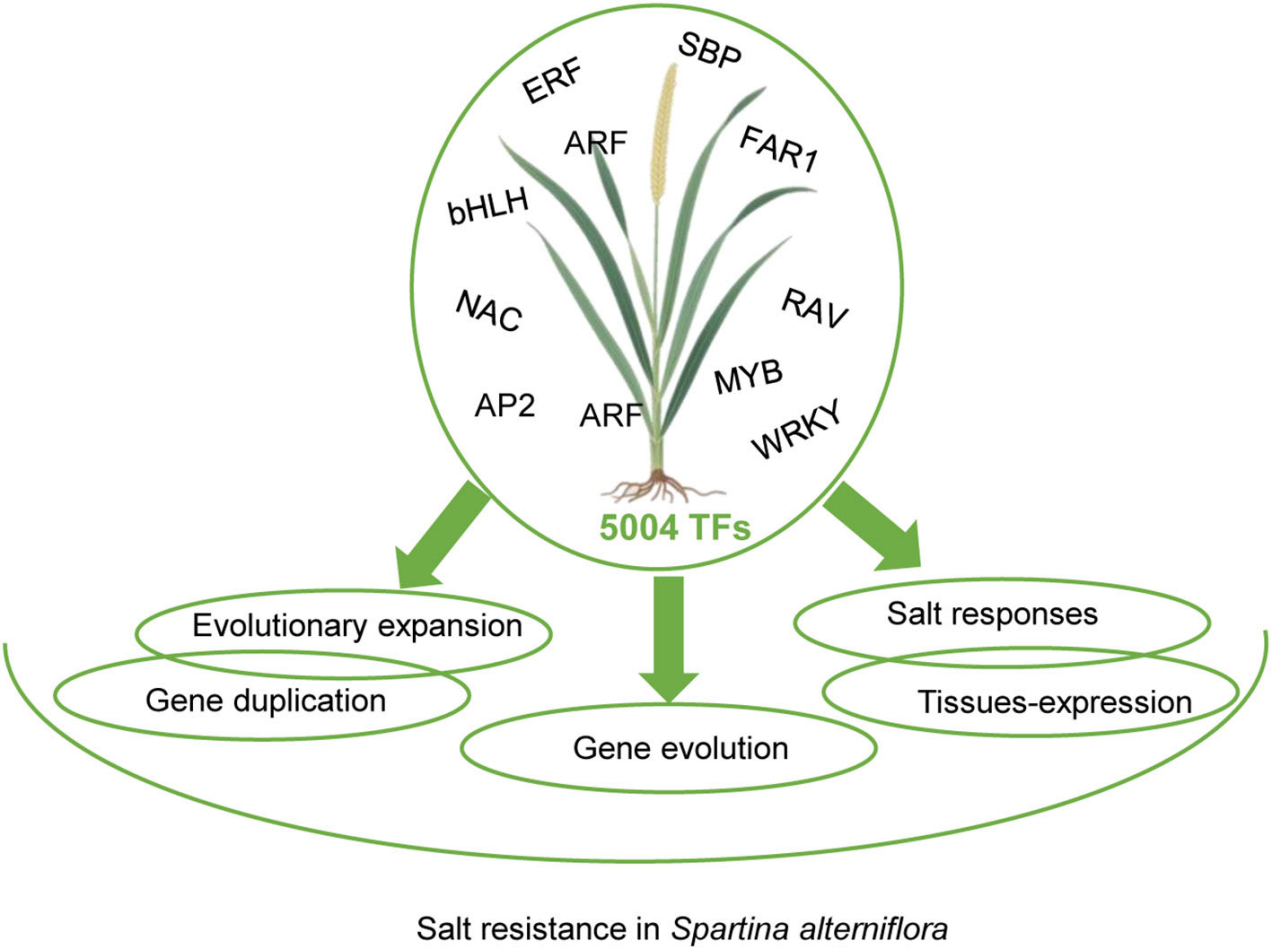
Proposed model illustrating the involvement of *Spartina alterniflora* transcription factors in salt stress resistance.

In summary, *S. alterniflora* employs TF family evolution, expansion, and functional diversification to achieve salt resistance, echoing strategies in model plants but with halophyte-specific innovations (**Figure 5**). Future work should: (i) functionally validate putative stress regulators (e.g., SaCPC1/2), (ii) resolve duplication-driven network rewiring, and (iii) compare TF regulatory hierarchies with glycophytes. These steps will bridge genomic observations to mechanistic physiology, advancing salt stress resilience in crops.

## 5 Conclusion

This study reveals the transcriptional regulatory basis of salt resistance in *S. alterniflora* through genome-wide TF analysis. We identified 5,004 TFs, notably enriched in bHLH, MYB, NAC, and ERF families, linked to transcriptional regulation and salt-stress pathways. Structural diversity and lineage-specific expansions via duplication events under purifying selection (Ka/Ks < 1) highlight functional diversification. Tissue-specific expression implicated MYB, WRKY, and bHLH in development, while salt stress induced 800 differentially expressed TFs, primarily from MYB, bHLH, and NAC families, regulating ion/osmotic homeostasis and antioxidant defense. These evolutionary and regulatory innovations underpin *S. alterniflora*’s ecological dominance in saline marshes, offering insights for crop resilience.

## Data Availability

RNA-seq data used in this study were obtained from the Bioproject accession numbers PRJNA949950, PRJNA949976, and PRJNA961432. All datasets analyzed in this study are publicly available, with repository names and accession numbers provided in the article and **Supplementary Material**.

## Supplementary material

Supplementary Figure 1 Phylogenetic comparison of **(A)** AP2, **(B)** ARF, **(C)** NAC, **(D)** RAV, (E) WRKY transcription factors (TFs) between *Spartina alterniflora* and rice, constructed using MEGA7. The shaded region illustrates a representative example of lineage-specific expansion within this TF family.

Supplementary Figure 2 Expression and KEGG enrichment analysis of root-specific cluster 12 genes under salt stress.
