# Supplementary material for: Characterization and expression analysis of transcription factors in Spartina alterniflora unveil their critical roles in salt stress resistance
Source: Front Plant Sci. 2025 Aug 21;16:1592211. doi: 10.3389/fpls.2025.1592211 (PMC12408496; doi:10.3389/fpls.2025.1592211)
Supplement: Supplementary Figure 1 — Phylogenetic comparison of (A) AP2, (B) ARF, (C) NAC, (D) RAV, (E) WRKY transcription factors (TFs) between Spartina alterniflora and rice, constructed using MEGA7. The shaded region illustrates a representative example of lineage-specific expansion within this TF family. [file DataSheet1.pdf]

A

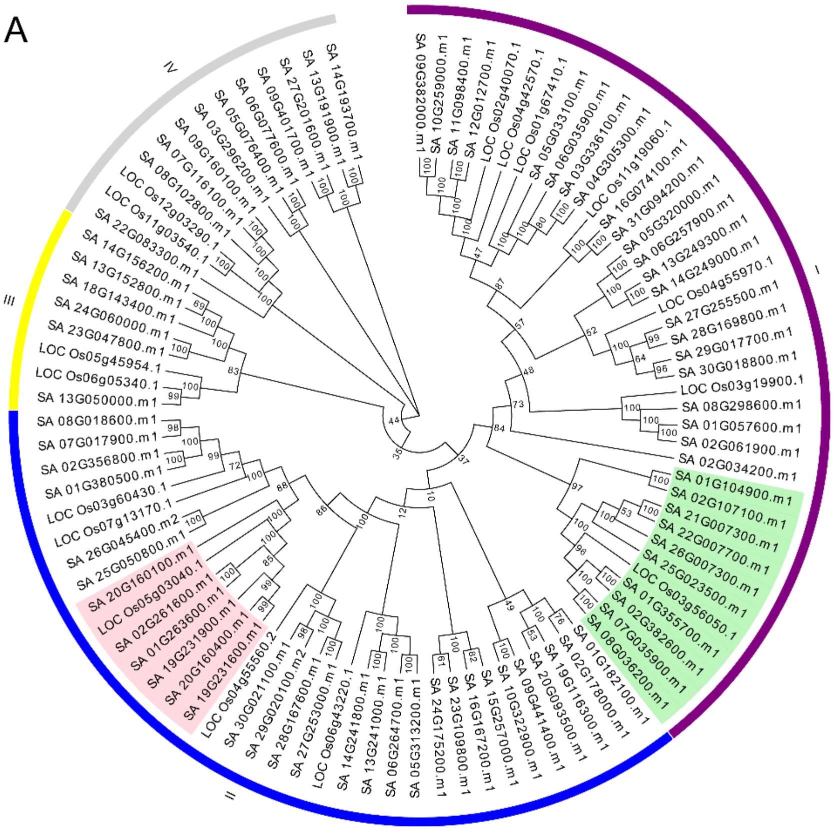
I
II
III
IV
SA 09G382000.m1
SA 10G259000.m1
SA 11G098400.m1
SA 12G012700.m1
LOC Os02g40070.1
LOC Os04g42570.1
LOC Os01g67410.1
SA 05G033100.m1
SA 06G035800.m1
SA 03G336100.m1
SA 04G305300.m1
LOC Os11g19060.1
SA 16G074100.m1
SA 31G094200.m1
SA 05G320000.m1
SA 06G257900.m1
SA 13G249300.m1
SA 14G249000.m1
LOC Os04g55970.1
SA 27G255500.m1
SA 28G169800.m1
SA 29G017700.m1
SA 30G018800.m1
LOC Os03g19900.1
SA 08G298600.m1
SA 01G057600.m1
SA 02G061900.m1
SA 02G034200.m1
SA 01G104900.m1
SA 02G107100.m1
SA 21G007300.m1
SA 22G007700.m1
SA 26G007300.m1
SA 25G023500.m1
LOC Os03g56050.1
SA 01G355700.m1
SA 02G382600.m1
SA 07G035900.m1
SA 08G036200.m1
SA 01G182100.m1
SA 02G178000.m1
SA 19G116300.m1
SA 20G093500.m1
SA 09G441400.m1
SA 10G322900.m1
SA 15G257000.m1
SA 16G167200.m1
SA 23G109800.m1
SA 24G175200.m1
SA 05G313200.m1
SA 06G264700.m1
SA 13G241000.m1
SA 14G241800.m1
LOC Os06g43220.1
SA 27G253000.m1
SA 28G167600.m1
SA 29G020100.m2
SA 30G021100.m1
LOC Os04g55560.2
SA 19G231600.m1
SA 20G160400.m1
SA 19G231900.m1
SA 01G263600.m1
SA 02G261600.m1
LOC Os05g03040.1
SA 20G160100.m1
SA 25G050800.m1
SA 26G045400.m2
LOC Os07g13170.1
LOC Os03g60430.1
SA 01G380500.m1
SA 02G356800.m1
SA 07G017900.m1
SA 08G018600.m1
SA 13G050000.m1
LOC Os06g05340.1
LOC Os05g45954.1
SA 23G047800.m1
SA 24G060000.m1
SA 18G143400.m1
SA 13G152800.m1
SA 14G156200.m1
SA 22G083300.m1
LOC Os11g03540.1
LOC Os12g03290.1
SA 08G102800.m1
SA 07G116100.m1
SA 09G160100.m1
SA 03G296200.m1
SA 05G076400.m1
SA 06G077600.m1
SA 09G401700.m1
SA 27G201600.m1
SA 13G191900.m1
SA 14G193700.m1

B

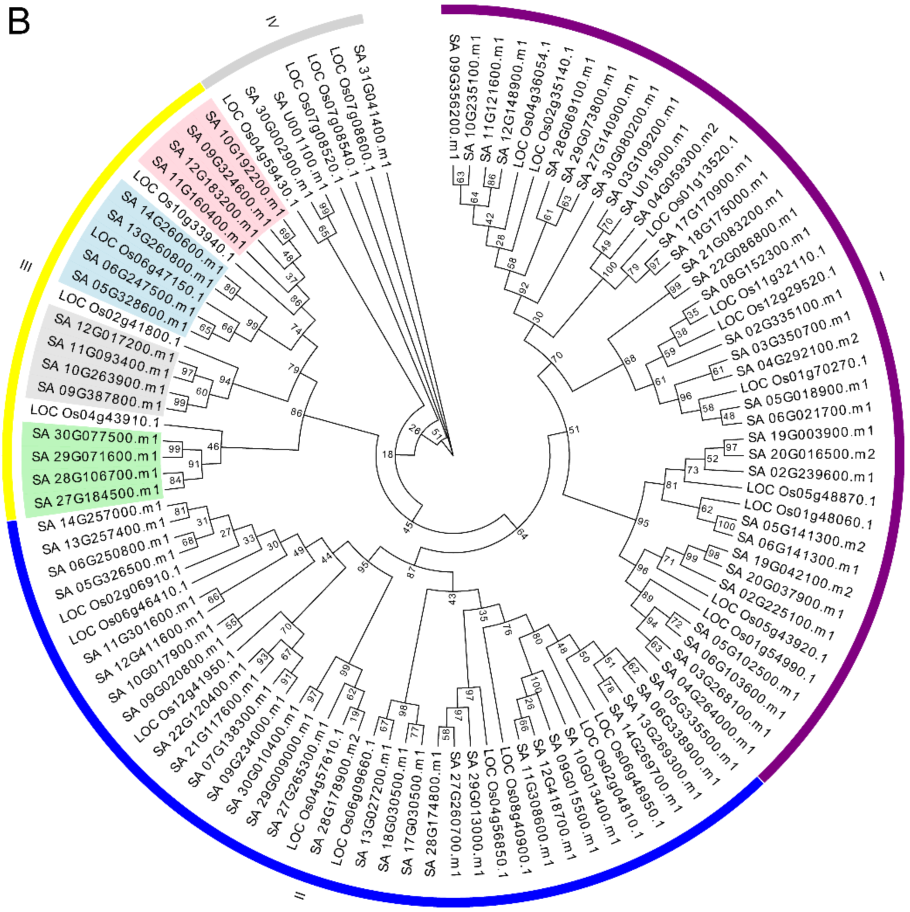
I
II
III
IV
SA 09G356200.m1
SA 10G235100.m1
SA 11G121600.m1
SA 12G148900.m1
LOC Os04g36054.1
LOC Os02g35140.1
SA 28G069100.m1
SA 29G073800.m1
SA 27G140900.m1
SA 30G080200.m1
SA 03G109200.m1
SA U015900.m1
SA 04G059300.m2
LOC Os01g13520.1
SA 17G170900.m1
SA 18G175000.m1
SA 21G083200.m1
SA 22G086800.m1
SA 08G152300.m1
LOC Os11g32110.1
LOC Os12g29520.1
SA 02G335100.m1
SA 03G350700.m1
SA 04G292100.m2
LOC Os01g70270.1
SA 05G018900.m1
SA 06G021700.m1
SA 19G003900.m1
SA 20G016500.m2
SA 02G239600.m1
LOC Os05g48870.1
LOC Os01g48060.1
SA 05G141300.m2
SA 06G141300.m1
SA 19G042100.m2
SA 20G037900.m1
SA 02G225100.m1
LOC Os05g43920.1
LOC Os01g54990.1
SA 05G102500.m1
SA 06G103600.m1
SA 03G268100.m1
SA 04G264000.m1
SA 05G335500.m1
SA 06G338900.m1
SA 13G269300.m1
SA 14G269700.m1
LOC Os08g48950.1
LOC Os02g04810.1
SA 10G013400.m1
SA 09G015500.m1
SA 12G418700.m1
SA 11G308600.m1
LOC Os08g40900.1
LOC Os04g56850.1
SA 29G013000.m1
SA 27G260700.m1
SA 28G174800.m1
SA 17G030500.m1
SA 18G030500.m1
SA 13G027200.m1
LOC Os06g09660.1
SA 28G178900.m2
LOC Os04g57610.1
SA 27G265300.m1
SA 29G009000.m1
SA 30G010400.m1
SA 09G234000.m1
SA 07G138300.m1
SA 21G117600.m1
SA 22G120400.m1
LOC Os12g41950.1
SA 09G020800.m1
SA 10G017900.m1
SA 12G411600.m1
SA 11G301600.m1
LOC Os06g46410.1
LOC Os02g06910.1
SA 05G326500.m1
SA 06G250800.m1
SA 13G257400.m1
SA 14G257000.m1
SA 27G184500.m1
SA 28G106700.m1
SA 29G071600.m1
SA 30G077500.m1
LOC Os04g43910.1
SA 09G387800.m1
SA 10G263900.m1
SA 11G093400.m1
SA 12G017200.m1
LOC Os02g41800.1
SA 05G328600.m1
SA 06G247500.m1
LOC Os06g47150.1
SA 13G260800.m1
SA 14G260600.m1
LOC Os10g33940.1
SA 11G160400.m1
SA 12G183200.m1
SA 09G324600.m1
SA 10G192200.m1
LOC Os04g59430.1
SA 30G002900.m1
SA U001100.m1
LOC Os07g08520.1
LOC Os07g08540.1
LOC Os07g08600.1
SA 31G041400.m1

C

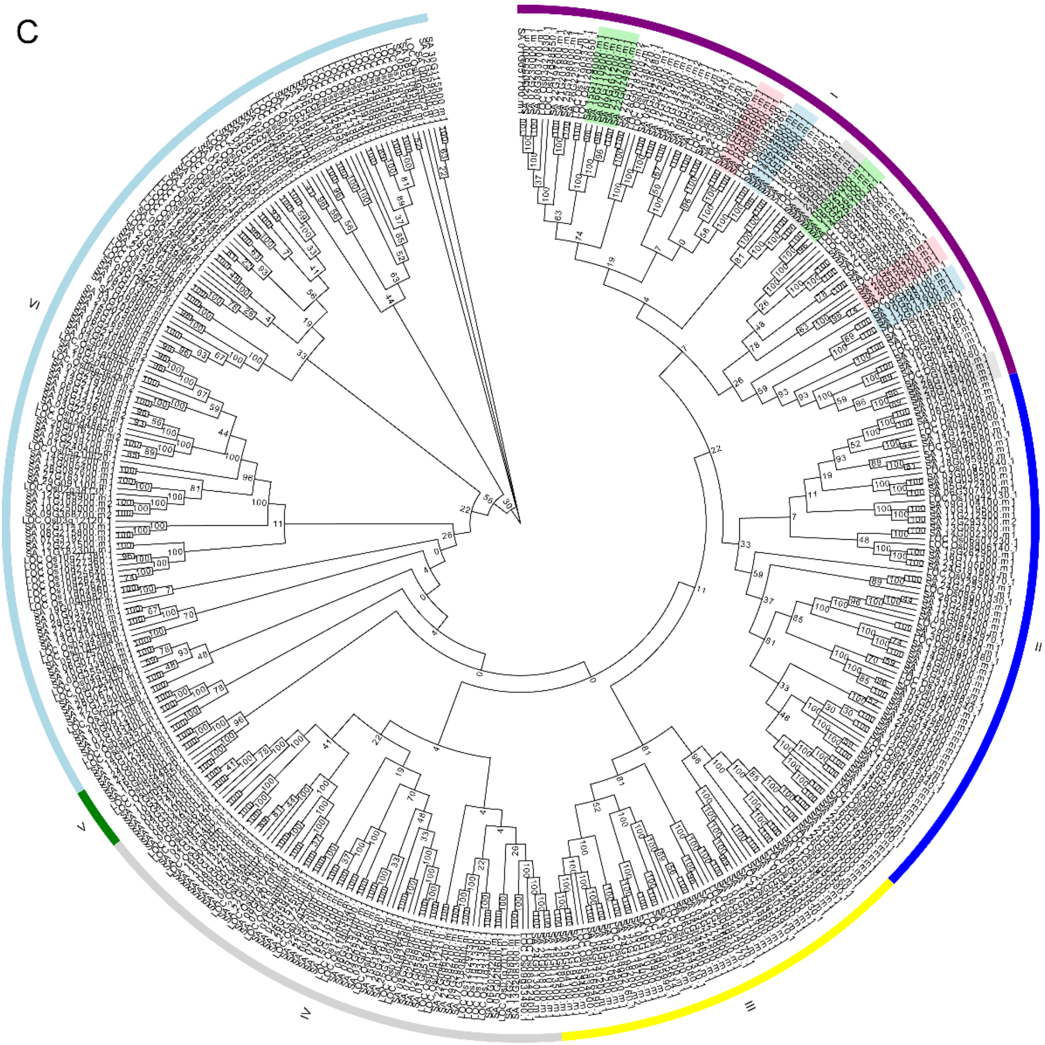

I
II
III
IV
V
VI

D

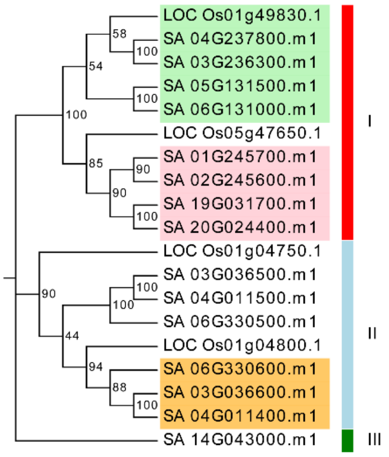

LOC Os01g49830.1
SA 04G237800.m1
SA 03G236300.m1
SA 05G131500.m1
SA 06G131000.m1
LOC Os05g47650.1
SA 01G245700.m1
SA 02G245600.m1
SA 19G031700.m1
SA 20G024400.m1
LOC Os01g04750.1
SA 03G036500.m1
SA 04G011500.m1
SA 06G330500.m1
LOC Os01g04800.1
SA 06G330600.m1
SA 03G036600.m1
SA 04G011400.m1
SA 14G043000.m1
I
II
III
58
100
54
100
100
85
90
90
100
100
100
90
44
94
88
100

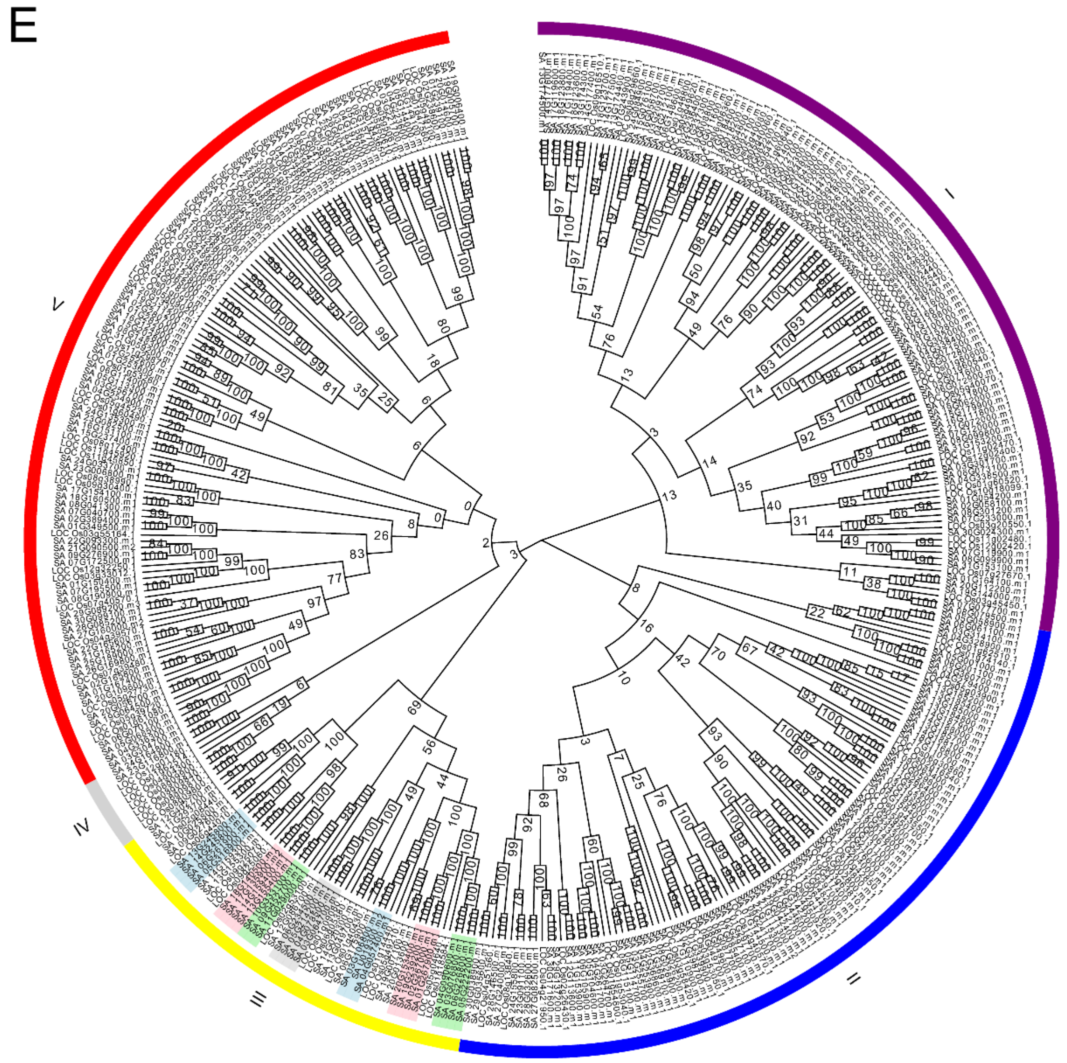

Figure S1. Phylogenetic comparison of (A) AP2, (B) ARF, (C) NAC, (D) RAV, (E) WRKY transcription factors (TFs) between *Spartina alterniflora* and rice, constructed using MEGA7. The shaded region illustrates a representative example of lineage-specific expansion within this TF family.
