# Supplementary material for: Characterization and expression analysis of transcription factors in Spartina alterniflora unveil their critical roles in salt stress resistance
Source: Front Plant Sci. 2025 Aug 21;16:1592211. doi: 10.3389/fpls.2025.1592211 (PMC12408496; doi:10.3389/fpls.2025.1592211)
Supplement: Supplementary Figure 2 — Expression and KEGG enrichment analysis of root-specific cluster 12 genes under salt stress. [file DataSheet2.pdf]

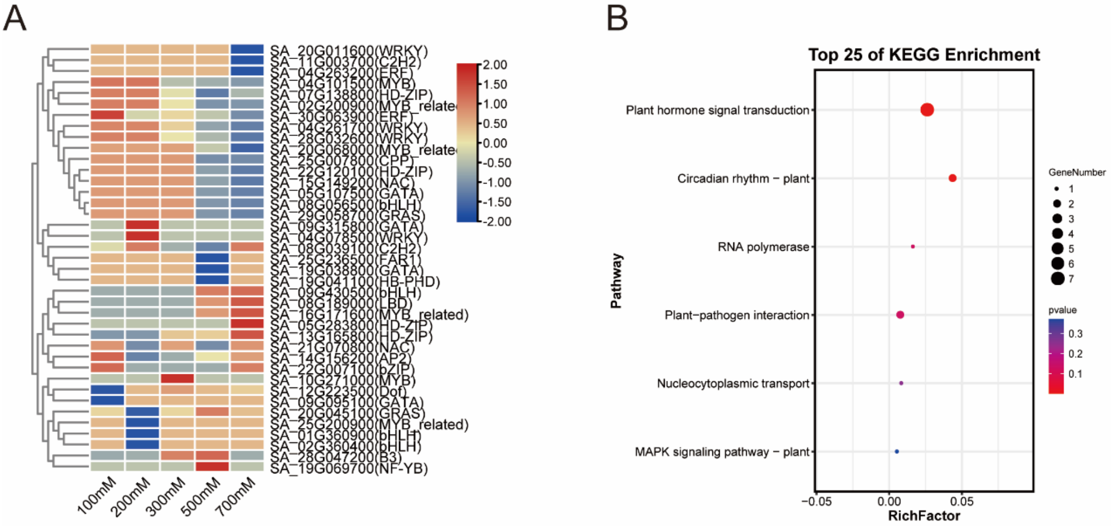

Figure S2. Expression and KEGG enrichment analysis of root-specific cluster 12 genes under salt stress.
